# Development of a novel expression system in lactic acid bacteria controlled by a broad-host-range promoter P_srfA_

**DOI:** 10.1186/s12934-022-01754-z

**Published:** 2022-02-15

**Authors:** Chengran Guan, Yuan Yuan, Yan Ma, Xin Wang, Chenchen Zhang, Maolin Lu, Ruixia Gu, Dawei Chen

**Affiliations:** 1 Key Lab of Dairy Biotechnology and Safety Control, College of Food Science and Technology, Yangzhou University, Yangzhou, 225127 Jiangsu China; 2 Shandong Yinfeng Life Science Research Institute, Jinan, 250000 Shandong China

**Keywords:** Broad-host-ranged promoter, Expression system, Lactic acid bacteria, Heterologous expression

## Abstract

**Background:**

Latic acid bacteria (LAB) are exploited for development of gene expression system owing to its health promoting properties and a high degree of safety status. Most of the expression systems were constructed in *Lactobacillus lactis* with inducible promoters. It is necessary to exploit novel promoters to develop LAB host platforms which are indispensable in dairy and health application to satisfy the production demand of increased number of target-genes. Previously, promoter P_srfA_ had been displayed broad host range and used to construct auto-inducible expression system in *B. subtilis* and *E. coli*. In this work, the feasibility of P_srfA_ in LAB was estimated.

**Results:**

Plasmid with the green fluorescent protein (GFP) inserting downstream of P_srfA_ was transformed into *L. casei* 5257, *L. plantarum* 97, *L. fermentum* 087 and *Weissella confusa* 10, respectively. The recombinant strains grew well and displayed different fluorescence which could be detected by spectrophotometer and laser scanning confocal microscope. Moreover, the promoter activity was strain- specifically influenced by particular carbon and nitrogen sources. Heterologous laccase CotA could be expressed by P_srfA_ in *L. casei* 5257-05 and *L. plantarum* 97-06. By adjusting the pH value from 4.5 to 6.5 during incubation, the CotA activity detected from *L. plantarum* 97-05 and *L. casei* 5257-05 was increased by 137.7% and 61.5%, respectively. Finally, the fermentation pH was variably up-regulated along with the production of NADH oxidase which was controlled by the P_srfA_ and its derivative mutated with core regions.

**Conclusions:**

These data suggested that P_srfA_ was valid for gene expression in different species of LAB. Moreover, P_srfA_ could be used as an attractive candidate for fine-tuning gene expression in a broad range of prokaryotic expression plants.

## Introduction

Expression host systems are developed for the expression of heterologous proteins and are becoming more and more attractive along with the development of molecular biology and the emergence of the synthetic biology [1]. So far, the extensively used expression systems were developed in *E. coli*, *Bacillus subtilis* and *Saccharomyces cerevisiae* due to their specific genetic background, mature genetic manipulation and capacity for continuous fermentation [2, 3]. The developed expression systems generally have inherent advantages and drawbacks, such as intracellular accumulation of heterologous proteins, lack of post-transcriptional modification, production of endotoxin, instability of plasmids and so on. It is necessary to exploit novel expression host platforms to satisfy the demand of producing the increased number of target-genes for various industrial productions.

Lactic acid bacteria (LAB) are a heterogeneous group of bacteria characterized by their ability to produce lactic acid as a fermentation product, including genera such as Lactococcus, Lactobacillus and Streptococcus. LAB are exploited for development of gene expression system owing to its health promoting properties and a high degree of safety status [4, 5]. Recently, remarkable achievements have been made towards the development of genetic engineering tools and therapeutic delivery systems to produce many different proteins of health interest, such as antigens, cytokines, vitamin, etc. [6–8]. The most successfully and widely used expression system is NICE (nisin controlled gene expression) system in *L. lactis* using promoter P_nisA_ which is proved to be a valuable tool for production of heterologous proteins including antimicrobial peptides, membrane proteins and vaccines [9]. Besides, some other inducible expression systems were constructed in LAB with promoters activated by lactose (P_lacA_), zinc (P_zn_), xylose (P_xylT_), fructo-oligosaccharide (P_fos_), etc. [10]. However, limited constitutive promoters were isolated which were able to be used for the production of food additives or application in the intestinal environment as LAB comprise a probiotic background. For example, promoter P_11_ (a synthetic sequence based on an rRNA promoter from *L. plantarum* WCSF1), promoter P_tuf33_ and P_tuf34_ (the promoter regions upstream of the gene encoding the putative translation elongation factor TU from *L. plantarum* CD033 and *L. buchneri* CD034) were demonstrated to be feasible for strong constitutive gene expression in *L. plantarum* [11].

The preferred expression system is selected depending upon the required quality and quantity of the target protein. Furthermore, the cost, availability and convenience of an optimal expression system should also be taken into consideration before making an informed choice of a system with which to express a foreign protein [12]. Therefore, it is necessary to evaluate the preliminary expression of the interest gene in the possible host cells. As the developed host cells have inherent genetic diversity, construction of cross-species expression system using the same genetic tools is a time- and cost-saving strategy to select the preferred expression cell for the target gene. The promoter, playing the role of expression switch, is commonly strain and context specific and can vary significantly within species. Hence, promoters available for a broad range of host strains should be developed to simultaneously examine gene expression in different genetic and physiological backgrounds and thus identify the most suitable expression strain. Only a limited number of promoters have been used in both Gram-positive and Gram-negative species, such as P_T7_, P_*xyl*_, P_*lac*_ and P_*BAD*_, etc. [13–15]. To date, several promoters with cross species activity were found in LAB. Promoter P_11_ was demonstrated to be component in *L. plantarum* and *L. sakei* and promoter P_tuf_ was highly active in some tested Lactobacillus strains as well as in *E. coli* [16].

Previously, the promoter of the *srf* operon (P_srfA_) originated from *B. subtilis*, had been used to construct expression systems in *B. subtilis* and *E. coli* in which heterologous proteins were highly produced without inducer [17, 18]. In this work, promoter P_srfA_ was used for construction of expression systems in LAB. The feasibility of promoter P_srfA_ was studied including the range of host strains, the factors affecting the promoter strength and the applicability of producing heterologous proteins. This work will enrich the efficient genetic toolbox for gene expression in LAB. Meanwhile, it will be helpful for cross-species gene expression or module construction in *E. coli*, *B. subtilis* and LAB.

## Results and discussion

### Feasibility of promoter P_srfA_ in different species of LAB

Hitherto, genetic engineering tools used for cloning heterologous genes in LAB have mainly exploited with *L. lactis* which has been used as the model microorganism in LAB research. Recently, some other LAB are identified to be indispensable in dairy and health applications which are potential to be used as production organisms for antimicrobials, exopolysaccharides, flavor-compound and texturing compound. With the increased interest in the probiotic function of the LAB, it is necessary to develop novel expression systems which could work in a broad range of LAB especially in the strains with proven probiotic [19]. Promoter P_srfA_, originated from *B. subtilis*, had already been used to develop expression system for production of heterologous proteins in *B. subtilis* and *E. coli* in our previous work [17, 18]. In this study, using the GFP as the report protein, the feasibility of P_srfA_ was evaluated in four representative genera of LAB which were wildly used in food production.

**Fig. 1:**
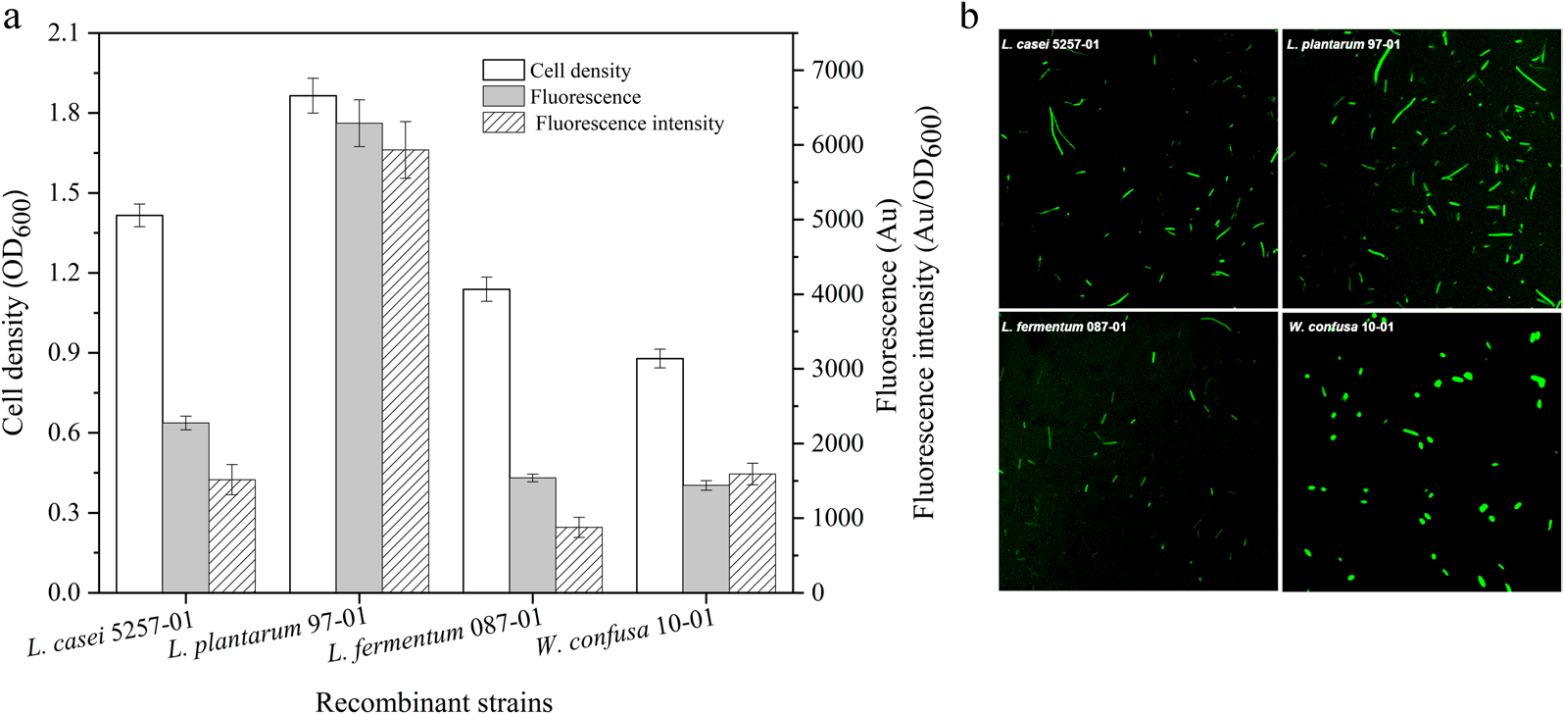
Expression of GFP controlled by P_srfA_ in recombinant strains (**a**) and fluorescence analyzed by laser scanning confocal microscope (**b**). Plasmid pMY01was transformed into *L. casei* 5257, *L. plantarum* 97, *L. fermentum* 087 and *W. confusa* 10, respectively. The cell growth, fluorescence and fluorescence intensity of the recombinant strains were detected

The recombinant plasmid pMY01 containing the fragment P_srfA_-*gfp* was separately transformed into *L. casei* 5257, *L. plantarum* 97, *L. fermentum* 087 and *W. confusa* 10, yielding the recombinant strain *L. casei* 5257-01, *L. plantarum* 97-01, *L. fermentum* 087-01 and *W. confusa* 10-01, respectively. After 24 h cultivation, the cell growth, the fluorescence and the fluorescence intensity (defined as fluorescence per biomass of OD_600_) were measured (Fig. 1). Although the wild strains were grew the same as the corresponding recombinant strains, there were no fluorescence to be detected (data not show). However, the recombinant strains displayed obviously different fluorescence (Fig. 1a). The highest fluorescence was obtained from *L. plantarum* 97-01 which was 1.76-, 3.08- and 3.23-times higher than that of *L. casei* 5257-01, *L. fermentum* 087-01 and *W. confusa* 10-01, respectively. The fluorescence intensity of *L. plantarum* 97-01 was 1.06- to 1.49-times higher than that of the other recombinant strains. Compared with the other three host strains, *L. plantarum* 97 was demonstrated to be most feasible to express GFP under the control of P_srfA_. It was equally shown in different studies that promoter strength was strain and context specific varied significantly within LAB [20]. Moreover, the recombinant strains with fluorescence could be obviously detected by laser scanning confocal microscope (Fig. 1b). These data suggested that promoter P_srfA_ had a broad transcriptional compatibility in different species of LAB.

### The effect of carbon and nitrogen sources on the activity of promoter P_srfA_

**Fig. 2:**
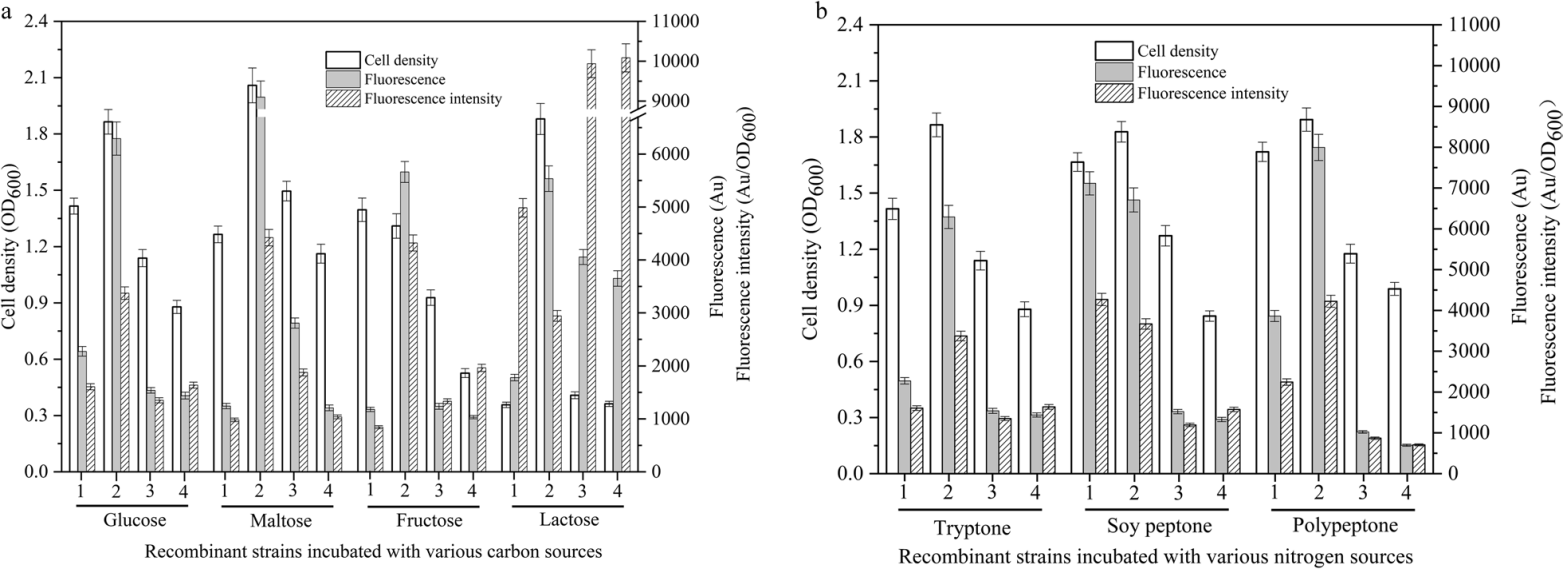
The effects of carbon (**a**) and nitrogen (**b**) sources on the biomass of recombinant strains and the activity of P_srfA_. The recombinant strains were individually cultivated in the medium containing various carbon and nitrogen sources. The strain biomass and expression of GFP were detected. 1: *L. casei* 5257-01, 2: *L. plantarum* 97-01, 3: *L. fermentum* 087-01, 4: *W. confusa* 10-01

The usual used carbon and nitrogen components for the cultivation of LAB were employed to estimated their effect on the expression of GFP controlled by P_srfA_. Firstly, the recombinant strains *L. casei* 5257-01, *L. plantarum* 97-01, *L. fermentum* 087-01 and *W. confusa* 10-01were cultivated in the medium containing glucose, maltose, fructose and lactose as the solo carbon source, respectively. The strain biomass was ranged from 0.36 (*W. confusa* 10-01 with lactose) to 2.09 (*L. plantarum* 97-01 with maltose) (Fig. 2 a). *L. plantarum* 97-01 showed better growth with these carbon sources than the other recombinant strains, especially than that of *W. confusa* 10-01. In addition, *L. casei* 5257-01 preferred glucose and fructose while *L. plantarum* 97-01, *L. fermentum* 087-01 and *W. confusa* 10-01 preferred maltose. Meanwhile, the fluorescence and the fluorescence intensity of recombinant strains were varied from 1032.95 Au (*W. confusa* 10-01 with fructose) to 9102.83 Au (*L. plantarum* 97-01 with maltose) and 843.96 Au/OD_600_ (*L. casei* 5257-01 with fructose) to 10,085.96 Au/OD_600_ (*W. confusa* 10-01 with lactose), respectively. Similarly, the recombinant strains were separately cultivated in the medium containing tryptone, soy peptone and polypeptone as the solo nitrogen sources. The particular recombinant strain showed consistent growth with the nitrogen sources. The strain biomass, fluorescence and the fluorescence intensity were ranged from 0.84 (*W. confusa* 10-01 with soy peptone) to 1.89 (*L. plantarum* 97-01 with polypeptone), 7996.13 Au (*L. plantarum* 97-01 with polypeptone) to 696.52 Au (*W. confusa* 10-01 with polypeptone) and 704.98 Au/OD_600_ (*W. confusa* 10-01 with polypeptone) to 4270.99 Au/OD_600_ (*L. casei* 5257-01 with soy peptone), respectively (Fig. 2b).

The varied fluorescence intensity was not directly proportional to the changed biomass among the recombinant strains with different carbon and nitrogen sources (Fig. 2). For instance, the biomass, fluorescence and fluorescence intensity of *L. plantarum* 97-01 cultivated with maltose were 0.63-, 6.33- and 3.50-times higher than that of *L. casei* 5257-01, respectively. In addition, the biomass, fluorescence and fluorescence intensity of *L. plantarum* 97-01 with maltose were 0.10-, 0.45- and 0.31-times higher than that of glucose. Moreover, to one specific stain, the difference of biomass and promoter activity with nitrogen sources were less than that of with carbon sources. Carbon and nitrogen components played different roles in the growth and survival of the bacteria, synthesis of enzymes and metabolites and regulation of the intracellular and extracellular environment, etc. All these factors directly or indirectly played different role in the activity of the promoter [21–23]. For instance, in *B. subtilis*, it was demonstrated that the activity of promoter P_srfA_ was negatively influenced by hydrogen peroxide (H_2_O_2_) because the H_2_O_2_ stress inactivated the DNA-binding activity of PerR which was required for activation of promoter P_srfA_ [24]. While in LAB, H_2_O_2_ is a highly relevant metabolite varied along with the carbon and nitrogen sources [25]. Therefore, carbon and nitrogen sources might affect the activity of promoter P_srfA_ by means of influencing H_2_O_2_ metabolism. These data suggested that the carbon and nitrogen source not only played an important role in the cell growth but also in the strength of the promoter P_srfA_.

### Application of promoter P_srfA_ in LAB

#### Expression of heterologous enzyme CotA originated from *B. subtilis*

**Fig. 3:**
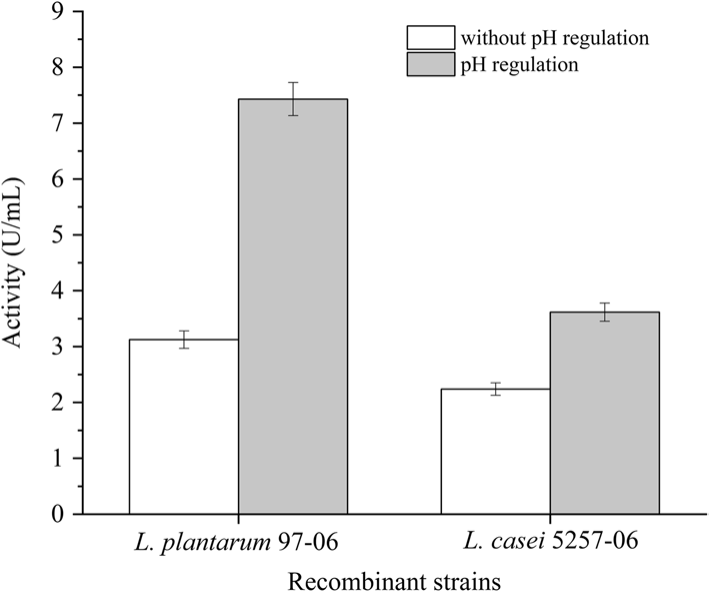
Expression of heterologous enzyme CotA controlled by P_srfA_. The recombinant strains containing CotA were incubated and the enzyme activity was detected. Further, the expression of CotA was tested by up-regulating the fermentation pH to 6.5 when the pH reduced below 4.5 during incubation

In spite of feasibility in various host strains, the other crucially important criteria for a mature expression system are the capacity to produce various target proteins [18]. Laccase represent an interesting group of oxidative enzymes among various enzymes owing to their great potential for biotechnological and environmental applications in biodegrading fields and food industry, such as wine stabilization, baking, etc. [26]. So far, laccase was mainly produced by the wild and recombinant fungus and there were scarce reports about laccase expression in LAB. Here, laccase (CotA), originated from *B. subtilis*, was used to estimate the suitability of P_srfA_ for the production of heterologous proteins in LAB. To express CotA, the gene fragment *cotA* was inserted downstream of P_srfA_ to replace *gfp* on plasmid pMY01. The yielded plasmid pMY05 was separately transformed into *L. plantarum* 97 and *L. casei* 5257 obtaining the recombinant strains *L. plantarum* 97-05 and *L. casei* 5257-05, respectively. After cultivation, the enzyme activity of CotA was detected. Compared with *L. plantarum* 97 and *L. casei* 5257, enzyme activity of CotA was obviously detected from *L. plantarum* 97-05 and *L. casei* 5257-05 (Fig. 3). The CotA activity of strain *L. plantarum* 97-05 was 0.40 times higher than that of *L. casei* 5257-05.

Moreover, by adjusting the pH of the medium to 6.5 when the pH reduced below 4.5 during incubation, the enzyme activity of *L. plantarum* 97-05 and *L. casei* 5257-05 was increased by 137.7% and 61.5%, respectively. Though there was enzyme activity, the corresponding protein band could not be detected on the SDS-PAGE. Considering on CotA and promoter P_srfA_ originated from *B. subtili*s, one explanation for the low expression might due to the different genetic background between Lactobacilus and Bacillus. Similarly, when nattokinase originated from *B. subtili*s was expressed in *L. lactis*, even though active nattokinase was markedly detected by western blotting and fibrinolytic activity analyses, it could not be detected with coomassie brilliant blue [27]. In addition, it was reported that various carbon and nitrogen sources were used to increase the production of laccase [28]. Therefore, to enhance the expression of laccase in LAB, the culture components and the strength of promoter P_srfA_ would be optimized in our subsequent work.

#### Expression of NADH oxidase to regulate the fermentation pH

Usually, LAB produce lactic acid during fermentation and the accumulated acid is a stress for the cell growth of LAB [29]. Normally, lactic acid is produced through glycolysis by converting pyruvate to lactate. Nevertheless, the pyruvate pool could be rerouted consequently through NADH-independent pathways when the intracellular pool of NADH was reduced by NADH oxidase (NOX) in the presence of oxygen. It was reported that when NOX was overexpressed, H^+^ was consumed as well as the reduced pyruvate to lactate flux was almost abolished [30]. Therefore, in this work, the pH of the supernatant was attempted to be regulated by the expression of NOX controlled by the promoter P_srfA_.

**Fig. 4:**
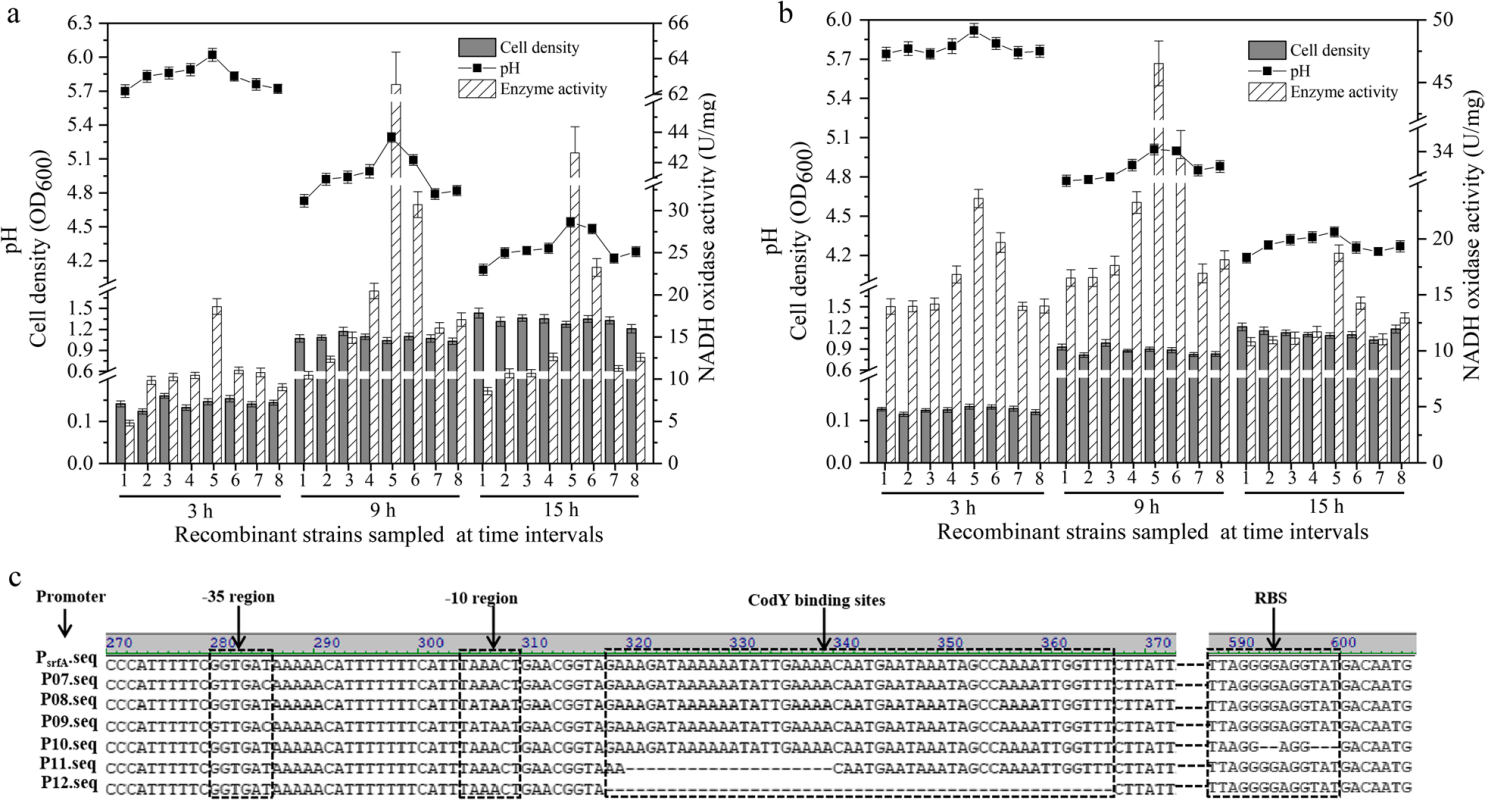
Regulation of the fermentation pH by production of NOX. The recombinant plasmids containing promoter P_srfA_ and its derivatives were transformed into *L. plantarum* 97 (**a**) and *L. casei* 5257 (**b**), respectively. The sequence of promoter P_srfA_ and its derivatives P07, P08, P09, P10, P11 and P12 which were mutated with -35 region, -10 region, -35 and -10 region, RBS, deletion of 20 bp of the CodY binding sites and deletion the entire region of Cody binding sites, respectively (**c**). The biomass, fermentation pH and NOX activity of the recombinant strains were detected at 3 h, 9 h and 15 h during fermentation. 1: wild strain, 2-8: recombinant strains containing promoter P_srfA_, P07, P08, P09, P10, P11 and P12, respectively

To express NOX, gene fragment *gfp* on the plasmid pMY01 was replaced by gene *nox*, obtaining plasmid pMY06. After transforming the pMY06 into *L. plantarum* 97 and *L. casei* 5257, the corresponding recombinant strains *L. plantarum* 97-06 and *L. casei* 5257-06 were cultured for 15 h as usual procedure. During fermentation, the recombinant strains grew similar to the wild-type strains and the NOX activity was detected from both the wild-type and recombinant strains (Fig. 4a, b). However, the NOX activity and fermentation pH measured from *L. plantarum* 97-06 were higher than that of *L. plantarum* 97 during fermentation (Fig. 4a). The NOX activity of *L. plantarum* 97-06 detected at 3 h, 9 h and 15 h was 1.03-, 0.18- and 0.24-times higher than that of *L. plantarum* 97, respectively. Meanwhile, the fermentation pH of *L. plantarum* 97-06 measured at 3 h, 9 h and 15 h was separately 0.13-, 0.19- and 0.15-∆pH higher than that of *L. plantarum* 97. These data suggested that gene *nox* was successfully expressed in *L. plantarum* 97 under the control of promoter P_srfA_ and the pH of the supernatant could be upregulated along with the expression of NOX. However, there was scarce difference of NOX activity and fermentation pH gap between *L. casei* 5257 and *L. casei* 5257-06 during incubation (Fig. 4b). It was speculated that gene *nox* expressed by promoter P_srfA_ in *L. casei* 5257 was too low to obviously affect the fermentation pH. The data indicated that the genetic background of *L. plantarum* 97 was more suitable than *L. casei* 5257 for promoter P_srfA_ to trigger the expression of NOX.

#### Fine-tunning the fermentation pH by promoter mutation

Metabolic engineering and synthetic biology approaches are becoming more and more attractive owing to its capacity to improve industrial application of microbes [1]. Generally, comparing with strong over-expression, strategies for the fine-tuning of gene expression are required to control the aimed metabolic fluxes in metabolic engineering. The most used method for accurate regulation of gene expression is promoter engineering by modifying the specific sequences of the promoter which is essential for the efficiency of transcription initiation and then for the expression of a targeted gene [30, 31]. To evaluate the validity of P_srfA_ for accurate gene expression, the sequence of P_srfA_ was analyzed. Besides the usual core regions of -10 box, -35 box and RBS (ribosome recognition site), there was a small sequence downstream of the promoter P_srfA_ which was probably the CodY binding site. CodY, a protein has proved to be ubiquitous in the low GC gram-positive bacteria and has been shown to regulate lots of genes expression by combining to target promoters to repress transcription of many genes while activate others [32]. Here, the conserved regions of the promoter P_srfA_ mentioned above were mutated using plasmid pMY06 as the template (Fig. 4c). The corresponding plasmids were individually transformed into *L. plantarum* 97 and *L. casei* 5257. The cell growth of the recombinant strains was similar to the wild strains (Fig. 4a, b). The NOX activity of the recombinant *L. plantarum* 97 containing the derivatives of P_srfA_ was varied from 9.02 U/mg to 62.55 U/mg which was accordingly 1.04- and 4.99-times higher than that of *L. plantarum* 97 during fermentation. Correspondingly, the pH of the supernatant detected from the recombinant strains was approximately 0.02- to 0.56-∆pH higher than that of the wide-type strain. Furthermore, the NOX activity and the fermentation pH of the recombinant strains containing the mutant promoters were diversely enhanced compared with that of P_srfA_. The mutant promoters P09 (mutations in -10 box and -35 box) and P10 (mutations in RBS region) could obviously enhance the enzyme expression and the fermentation pH of the corresponding recombinant strains was markedly up-regulated. The promoter P08 ((mutations in -35 box) showed higher enzyme expression than the promoter P07 ((mutations in -10 box). One of the explanation might be the identity of the original sequence of -10 box (TAAACT) with the prokaryotic conserved -10 region (TATAAT) was higher than the original -35 box (GGTGAT) with the prokaryotic conserved -35 box (TTGACA). When combining the mutations in -10 and -35 regions together (promoter P09), the NOX activity controlled by promoter P09 was 3.19- and 2.05-times higher than that of promoters P07 and P08, respectively. Besides, the promoter P10 with mutation in the RBS region showed brilliant performance of NOX expression and pH up-regulation. It was suggested that the optimized RBS sequence provided optimal complementation to the 3′-end of the 16S rRNA, thereby increasing the ribosome’s affinity to the mRNA and enhancing the translation initiation efficiency. In addition, the promoters P11 and P12 mutated with the predicted CodY binding site showed slight higher NOX activity than that of promoter P_srfA_, suggesting that the CodY protein in LAB was likely to repress the activity of promoter P_srfA_. Currently, despite the detailed studies on CodY-mediated regulation, which revealed direct interaction between CodY and the regulatory regions of its targets, no CodY recognition sequence could be deduced [33]. Hence, the enhanced activity of the mutated promoter P11 and P12 might have no relation with CodY. It was supposed that the truncation of the predicted CodY binding sites was helpful for the RNA polymerase and the mutated promoters to generate a more stable topological structure and then strengthen the transcription. These predictions need to be verified in our followed work. Likewise, Guo constructed a constitutive promoter library by randomizing the -35 and -10 region of the promoter sequence of the *noxE* gene (H_2_O-forming NADH oxidase) in *L. lactis* and the fermentation pH was fine-tunned ranging from 5.93 to 6.32 [30]. Furthermore, the similar varied trend of enzyme activity and the supernatant pH were also found in the recombinant host strains *L. casei* 5257. These data demonstrated that the core regions of P_srfA_ were valid in LAB exhibiting different promoter strength and were able to regulate the fermentation pH by governing the expression of NOX.

The main focus of metabolic engineering has been on *E. coli* and *S. cerevisiae* as microbial workhorse. Recently, LAB has gained increasing attention because of its indispensable in dairy and health applications. Firstly, many engineering efforts were made concentrating on *L. lactis* in which the glycolytic highway and its uncoupling of other catabolic pathways were efficiently rerouted resulting in the high level production of both natural and novel end products, such as vitamin, polyol, extracellular polymeric substances, etc. [34]. But there were only a few genetic tools developed in other LAB except *L. lactis* which were used in probiotic products. Secondly, most expression system developed in LAB were using inducible promoters, such as P_nisA_, P_zn_, P_lac_, P_170_ which were induced by nisin, heavy metals, sugar and pH, respectively [35–38]. Inducible expression can be preferable in cases where the aim is to overproduce a desired protein at high levels, e.g. at a specific moment during fermentation. However, as LAB comprise a food grade background, inducible systems are less suitable in the intestinal environment or when steady-state gene expression is required, instead, constitutive promoters providing expression of a target gene at a suitable level are desirable [39]. In addition, constitutive promoters are preferred to construct synthetic promoter library for the fine-tuning of gene expression which is important for applications as metabolic optimization and metabolic control analysis [40].

In general, promoter P_srfA_ used in this work was able to constitutively trigger gene expression at distinct times during growth in different species of LAB. Moreover, P_srfA_ had been proved to be valid to express various heterologous proteins in *B. subtilis* and *E. coli*. Thus, promoter P_srfA_ was feasible for the construction of circuits functional in multiple species as well as convenient preliminary screening of before deployment in *E. coli*, *Lactobacillus spp.* or *Bacillus spp*.

## Conclusion

Developing of genetic engineering tools for gene expression have been mainly made in *L. lactis* and the limited *Lactobacillus spp*. In this work, promoter P_srfA_ originated from *B. subtilis*, was verified to be valid to trigger the expression of GFP and heterologous protein CotA in different species of LAB. Moreover, P_srfA_ and its derivatives were able to accurately tune the fermentation pH by managing the production of NOX. Besides, in our previous work, promoter P_srfA_ was available to produce heterologous proteins in different genus of *B. subtilis* and *E coli.* Hence, promoter P_srfA_ could be used as an attractive candidate in metabolic engineering for tuning gene expression in broad range of prokaryotic expression plants.

## Materials and methods

### Strains, plasmids and incubated conditions

*E. coli* JM109 was used as a host for gene cloning. *L. casei* 5257, *L. plantarum* 97, *L. fermentum* 087 and *Weissella confusa* 10 were used for the gene expression. The recombinant strains and plasmids used in this study are listed in Table 1. The vector pColdII-Z containing gene *cot*A for enzyme Laccase was kindly given by Zhang YZ. (Yancheng Institute of Technology, Jiangsu, China). The media used for *E. coli* and LAB were LB (Luria–Bertani) medium and MRS (de Man, Rogosa and Sharp) medium. When appropriate, growth media for recombinant strains were supplied with ampicillin (10 μg/mL) and chloramphenicol (10 μg/mL).

**Table 1:** Recombinant strains and plasmids used in this study

Recombinant strains and plasmids	Characteristics	Reference
Recombinant strains		
*L. casei* 5257-01	*L. casei* 5257 with plasmid pMY01	This work
*L. plantarum* 97-01	*L. plantarum* 97 with plasmid pMY01	This work
*L. fermentum* 087-01	*L. fermentum* 087 with plasmid pMY01	This work
*W. confusa* 10-01	*W. confusa* 10 with plasmid pMY01	This work
*L. plantarum* 97-05	*L. plantarum* 97 with plasmid pMY05	This work
*L. plantarum* 97-06	*L. plantarum* 97 with plasmid pMY06	This work
*L. plantarum* 97-07 to *L. plantarum* 97-12	*L. plantarum* 97 with plasmid pMY07 to pMY12, respectively	This work
*L. casei* 5257-05	*L. casei* 5257 with plasmid pMY05	This work
*L. casei* 5257-06	*L. casei* 5257 with plasmid pMY06	This work
*L. casei* 5257-07 to *L. casei* 5257-12	*L. casei* 5257 with plasmid pMY07 to pMY12, respectively	This work
Plasmids		
pMY01 pColdII-Z pMY05 pMY06 pMY07 pMY08 pMY09 pMY10 pMY11 pMY12	pNE8148 with P_nisA_ replaced by the fragment of P_srfA_-*gfp* pColdII with gene *cotA* pMY01 with gene *gfp* replaced by gene *cotA* pMY01 with gene *gfp* replaced by gene *nox2* pMY06 containing promoter P07 (-35 region of P_srfA_ mutated into TTGACA) pMY06 containing promoter P08 (-10 region of P_srfA_ mutated into TATAAT) pMY06 containing promoter P09 (-35 and -10 regions of P_srfA_ mutated into TTGACA and TATAAT) pMY06 containing promoter P10 (the RBS region of P_srfA_ mutated into TAAGGAGG) pMY06 containing promoter P11 (deletion 20 bp of CodY binding sequence in P_srfA_) pMY06 containing promoter P12 (deletion CodY binding sequence in P_srfA_)	This work This work This work This work [18] This work This work This work This work

### Recombinant DNA techniques

Plasmid construction was performed in *E. coli* and DNA extraction was performed by following a standard procedure as previously described [17]. Recombinant plasmids were transformed into LAB as previously described [16]. The primers used in this study are listed in Table 2. PCR was performed using primerSTAR max DNA polymerase (Takara, Japan). All of the recombinant plasmids constructed in this work were confirmed by DNA sequencing (Shanghai Sangon Biotech Co., Ltd., China).

**Table 2:** Sequence of primers used in this study

Primers	Sequence
F1 R1 F5 R5 F6 R6 F7 R7 F8 R8 F9 R9 F10 R10 F11 R11 F12 R12	CATTGTCATACCTCCCCTAAT GGCACTCACCATGGGTACT GCTTATAAAGATTAGGGGAGGTATGACAATGATGAACCTAGAAAAGTTTG GCATGCCTGCAGTACCCATGGTGAGTGCCCTAAATGATATCCATCGGCC AAAGATTAGGGGAGGTATGACAATGATGAAAGTTGCCATTATTGG TGCCTGCAGTACCCATGGTGAGTGCCTTAATTATTCCGACGATAGC TGAAACTTTTCACCCATTTTTCGTTGACAAAAACATTTTTTTCATT CAGTTTAAATGAAAAAAATGTTTTTGTCAACGAAAAATGGGTGAAAAG GGTGATAAAAACATTTTTTTCATTTATAATGAACGGTAGAAAGAT TATTTTTTATCTTTCTACCGTTCATTATAAATGAAAAAAATGTTT TGAAACTTTTCACCCATTTTTCGTTGACAAAAACATTTTTTTCATT CATTATAAATGAAAAAAATGTTTTTGTCAACGAAAAATGGGTGAAAAG GCACATGTTCACTGCTTATAAAGTAAGGAGGTATGACAATGATGAAAG GGCAACTTTCATCATTGTCATACCTCCTTACTTTATAAGCAGTGAAC TTCATTTAAACTGAACGGTAAACAATGAATAAATAGCCAAAATTGGTT TTGGCTATTTATTCATTGTTTACCGTTCAGTTTAAATGAAAAAAATGT TTCATTTAAACTGAACGGTATCTTATTAGGGTGGGGTCTTGCGGTCT AAGACCCCACCCTAATAAGATACCGTTCAGTTTAAATGAAAAAAATGT

Plasmid pMY01 was linearized using inverse PCR with primers F1 and R1. Gene *cotA* flanked by a 30-bp homology sequence upstream and downstream of the inserted position in pMY01 was amplified from plasmid pColdII using primers F5 and R5. The purified linearized pMY01 and *cotA* fragment were connected according to the manufacturers’ protocols of the Clone ExpressTM II One Step Cloning Kit (Vazyme Biotech Co., Ltd, Nanjing, China). The product was transformed into *E. coli* and the sequenced clone was named as pMY05. Plasmid pMY06 was constructed according to the steps described above. Gene *nox* was cloned from *L. plantarum* genome using primers F6 and R6 which was then connected with the purified linear pMY01, yielding plasmid pMY06.

To construct the plasmids containing P_srfA_ with mutation in the core region, plasmid pMY06 was used as the template by means of a megaprimer PCR as previously described [18]. The derivatives of pMY06 (from pMY07 to pMY12 containing the related promoters P07–P12) with mutation in the core sequences of the promoter were obtained with the corresponding primers.

### Cultivation of LAB

A single colony of the appropriate recombinant LAB strain was picked from an MRS agar plates, inoculated in 10 mL of MRS liquid medium and cultured at 37 °C for 24 h. Then, 0.3 mL of the culture were inoculated in 10 mL of MRS liquid medium and cultured at 37 °C for 12 h to serve as a preculture. Then, 6 mL of the preculture was transferred to 250-mL flasks that contained 200 mL of MRS liquid medium and cultivated at 37 °C for 24 h during which the culture medium was sampled with time intervals. After cultivation, the cells and the supernatant were separated by centrifugation. The supernatant was used for pH detection. The harvested cells were washed by PSB buffer (50 mM Tris–HCl, 100 mM NaCl, pH 7.5) for three times, and suspended in an appropriate diluted ratio to be used for detection of fluorescence and enzyme activity.

### Detection of cell growth and pH

The cell density was determined by measuring the OD600 with a UV-1800/PC spectrophotometer (MAPADA Instrument Co., Shanghai, Ltd., China). The pH was measured using a laboratory pH meter (Mettler Toledo FE20, Shanghai, China). Each trial was performed in triplicate.

### Effect of carbon source, nitrogen source and pH

To analyze the effect of carbon and nitrogen sources, MRS medium with glucose was separately replaced by the equal weight of maltose, fructose and lactose while tryptone replaced by equal weight of soy peptone and polypeptone, respectively. To assay the effect of the pH, sodium hydroxide solution (3 mol/L) was used to control the pH. The strains were cultivated in the MRS medium with the initial pH of 6.5, 7.5, 8.0 and 9.0 to analyze the effect of the initial pH. Besides, to evaluate the effect of the pH during fermentation, the strains were cultivated in the MRS medium and then the pH were separately regulated to 5.5, 6.5 and 7.5 when the pH declined to 4.5 during cultivation. For the above cultivation, assays were done with the usual procedure.

### Assay of GFP fluorescence

The fluorescence and the image of the harvested cells were separately detected by spectrophotometer and laser scanning confocal microscope as previously described [18]. All the data were averaged from three independent samples of the same time points.

### Assay for NADH oxidase

Three milliliters of the preculture of the suitable strains were incubated in a 250-mL flask loading 100 mL MRS medium and cultivated in a shaker incubator at rotate speed of 120 r/min at 37 °C for 15 h. The sample was obtained at 3 h, 9 h and 15 h. After centrifugation, the supernatant and the cells were used for detection of pH and the activity of NADH oxidase, respectively. The harvested cells were disrupted on ice by a cell disruptor (Constant System Ltd., England) and then centrifugated for 10 min (4 °C, 10,000 r/min). The corresponding supernatant was used for the detection of the protein concentration and the enzyme activity according to the instruction of the NADH oxidase test kit and total protein quantitative assay kit (Jiancheng Bioengineering Institute, Nanjing, China). One unit of activity was defined as the absorbance increase equal to 0.01 in 1 min at 600 nm per milligram protein. The results are the averages of triplicate assays.

## Data Availability

All data used this study are included in this published article.
